# Supplementary material for: Circulating androgen regulation by androgen-catabolizing gut bacteria in male mouse gut
Source: Gut Microbes. 2023 Feb 26;15(1):2183685. doi: 10.1080/19490976.2023.2183685 (PMC9980454; doi:10.1080/19490976.2023.2183685)
Supplement: Supplemental Material [file KGMI_A_2183685_SM6618.zip › ID222204625_R1_Mouse_Gut_Thauera_Supplemental_Information.pdf]

Supplementary Information for

Circulating androgen regulation by androgen-catabolizing gut bacteria in male mouse gut

Tsun-Hsien Hsiao, Chia-Hong Chou, Yi-Lung Chen, Po-Hsiang Wang, Guo-Jie Brandon-Mong, Tzong-Huei Lee, Tien-Yu Wu, Po-Ting Li, Chen-Wei Li, Yi-Li Lai, Yu-Lin Tseng, Chao-Jen Shih, Po-Hao Chen, Mei-Jou Chen, and Yin-Ru Chiang

Correspondence to Yin-Ru Chiang and Mei-Jou Chen
Email: yinru915@gate.sinica.edu.tw; mjchen04@ntu.edu.tw

This PDF file includes:

Supplemental Results
Figures S1 to S8
Table S1 to S3
Legends for Datasets S1 and S2
Appendices S1 to S4

Other supplementary materials for this manuscript include the following:

Dataset S1
Dataset S2

Supplemental Results

Genomic and transcriptomic analyses of strain GDN1

We obtained the whole genome sequence of strain GDN1 through the PacBio sequencing. The 3.87-Mb chromosome (68% G + C; accession number: CP097870) contains 3590 genes, including 3488 of predicted protein-coding genes, 65 of tRNA genes, 4 copies of rRNA operons (5S, 16S and 23S) and 25 genes coding for other RNA products (**Dataset S1**). Consistent with the observed phenotype, strain GDN1 contains a complete set of genes involved in denitrification and tricarboxylic acid cycle (**Fig. S6**). Gene cluster responsible for fatty acid metabolism through β-oxidation reactions (CKCBHOJB_03119~_03124) was identified. However, only a few genes participating in chemotaxis and flagellum biosynthesis were identified in the strain GDN1 genome, suggesting the minor role of chemotaxis in bacterial androgen catabolism.

The strain GDN1 genome contains a complete set of androgen catabolic genes participating in the established anaerobic steroid 2,3-*seco* pathway [30] and aerobic steroid 9,10-*seco* pathway, including those involved in aerobic steroid A/B-ring catabolism (Locus tags: CKCBHOJB_02409 to 02411, CKCBHOJB_01547 to 01548, CKCBHOJB_02256, CKCBHOJB_02263 to 02265, CKCBHOJB_02279, CKCBHOJB_02322, CKCBHOJB_02329, CKCBHOJB_02331 to 02332, CKCBHOJB_02374 to 02375, and CKCBHOJB_02377 to CKCBHOJB_02381) and C/D-ring catabolism (CKCBHOJB_02283 to 02287, CKCBHOJB_02313 to 02321, CKCBHOJB_02328, and CKCBHOJB_02372) (**Dataset S1**). Among them, the 3-ketosteroid 9α-hydroxylase gene (*kshAB*; CKCBHOJB_02279 and _02264; **Dataset S1**) is responsible for the O_2-dependent cleavage of the androgen B-ring. On the other hand, the gene products (namely the bifunctional 1-testosterone hydratase/dehydrogenase) of *atcABC* (CKCBHOJB_02409 to 02411; **Dataset S1**) mediate the O_2-independent androgenic A-ring activation by adding a hydroxyl group at C-1 of testosterone [30]. Thus far, most genes involved in the anaerobic catabolism of steroid A/B-rings have not been identified. Both the aerobic and anaerobic androgen catabolic pathway are channeled into a common intermediate, 3aα-H-4α(3'-propanoate)-7aβ-methylhexahydro-1,5-indanedione (HIP); the carboxylic acid with the remaining steroid C/D-rings is further catabolized through the same set of bacterial genes, regardless of oxygen availability. The genes involved in the HIP catabolism could be identified in the strain GDN1 genome (**Fig. 3**).

To investigate the expression of androgen catabolic genes of strain GDN1, strain GDN1 cells were grown under several growth conditions, including aerobic growth with acetate, aerobic growth with testosterone, anaerobic growth with acetate, and anaerobic growth with testosterone. The growth experiments are conducted in duplicate, and the strain GDN1 transcriptomes were sequenced on an Illumina platform. Paired-end reads obtained from four treatments have been deposited in the NCBI SRA database [BioProject ID PRJNA838737 (accession numbers: SRR19418054~ SRR19418057)]. The unbiased distribution of the selected housekeeping genes (see **Table S3** for detailed information on individual genes) indicated the continuous expression of these housekeeping genes among active strain GDN1 cells, regardless of growth conditions. By contrast, most of the androgen catabolic genes were only apparently expressed in the testosterone-grown cells (**Fig. 3*B***; left panel). However, these androgen catabolic genes are still expressed at a low level by the acetate-grown strain GDN1 (**Fig. 3**). Surprisingly, similar gene expression patterns are observed in the strain GDN1 cells grown aerobically or anaerobically with testosterone (**Fig. 3*B***; right panel). Moreover, the transcriptomic analysis indicated that genes corresponding to the 2,3-*seco* pathway, namely *atcABC*, were still apparently expressed by the aerobically testosterone-grown cells, while genes corresponding to the oxygenase-dependent 9,10-*seco* pathway (*e.g., kshAB*) were apparently expressed in the anaerobic strain GDN1 cultures. The

indistinguishable expression of the androgen catabolic genes in the anaerobically and aerobically testosterone-grown cultures indicated that these catabolic genes are induced by the substrate but not oxygen conditions. Together, our transcriptomic analysis suggested the expression of both aerobic and anaerobic androgen catabolic genes in the O_2-limited mouse gut. The transcriptomic data also revealed the conspicuous expression (fold changes > 4) of numerous transporter genes (CKCBHOJB_00171, _00680, _02330, _02356, _02365, _02382, _02389, _02470, _02975, and _03302) in the testosterone-grown cultures. The role of these transporters in androgen catabolism remains to be investigated at a molecular level.

Supplemental Figures

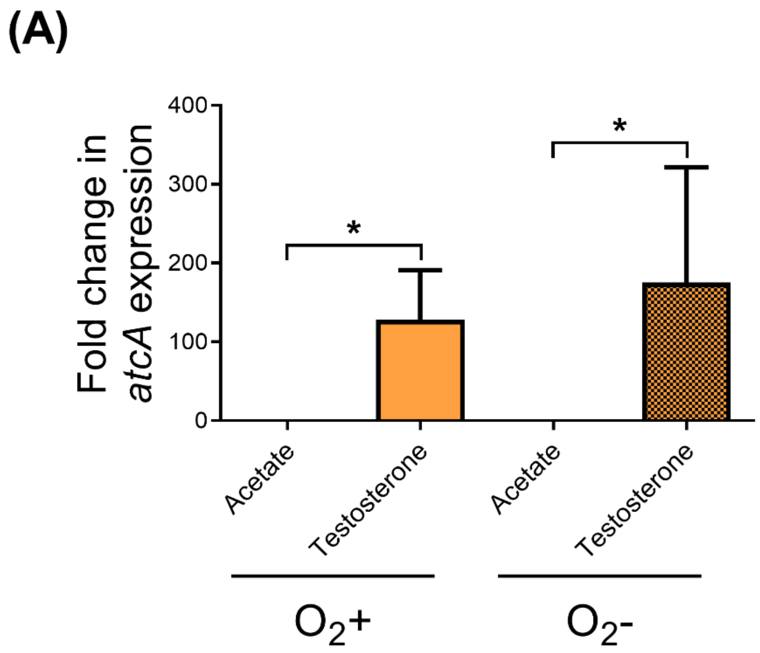

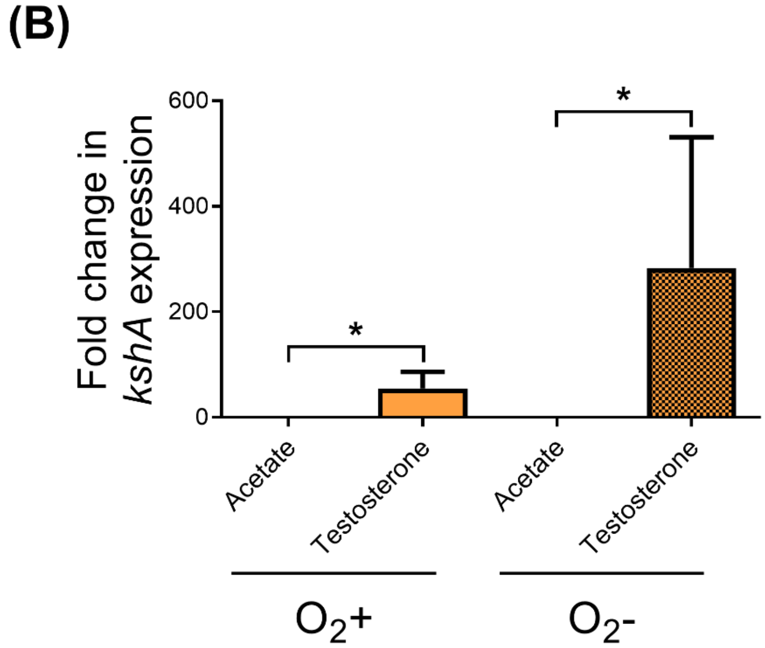

Fig. S1. Determination of relative gene expression of the strain GDN1 *atcA* (**A**) and *kshA* (**B**) using RT-qPCR. *Thauera* sp. strain GDN1 was grown with acetate or testosterone as the sole carbon source under aerobic or anaerobic (denitrifying) conditions. Statistical results were calculated with unpaired nonparametric *t*-test; *$p < 0.05$. All data are shown as means ± SEM.

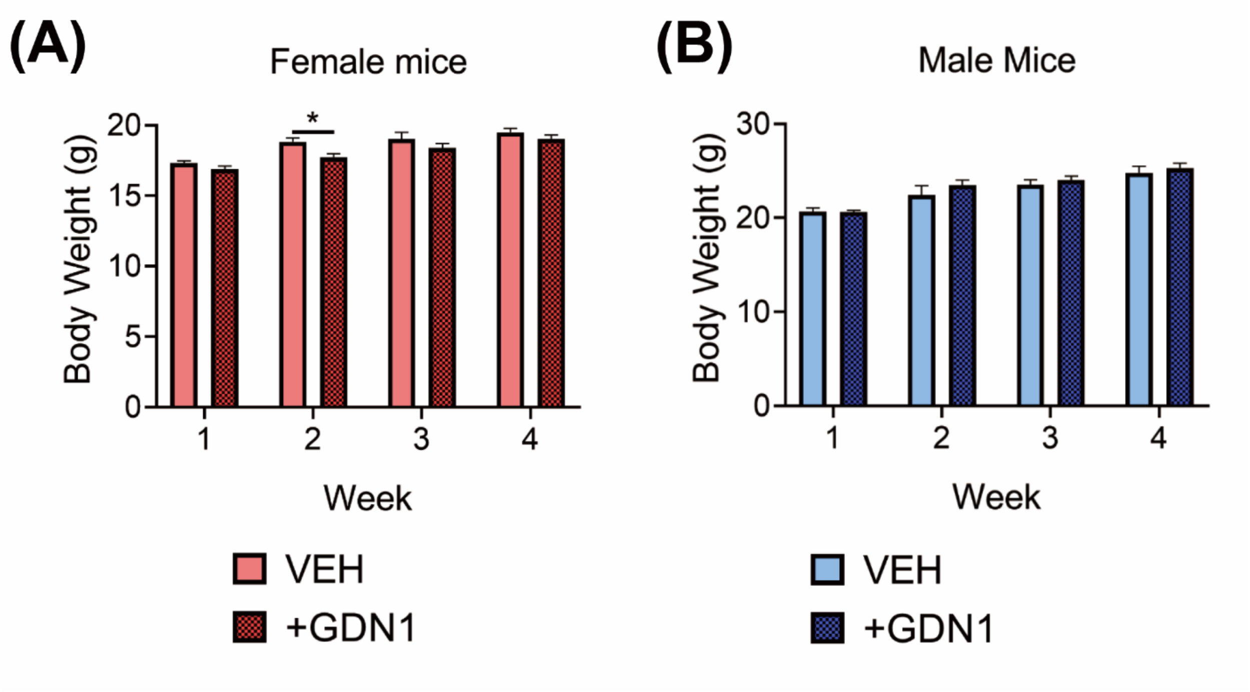

Fig. S2. Temporal changes in body weight of the tested female (**A**) and male (**B**) mice. Statistical results were calculated with unpaired *t*-test; $*p < 0.05$. All data are shown as means ± SEM from 8–14 mouse individuals.

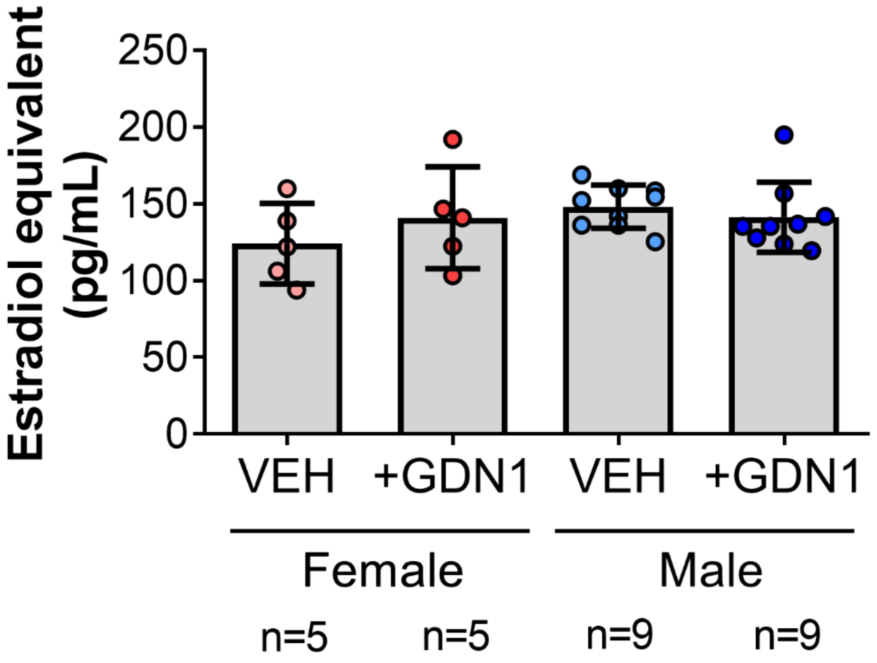

Fig. S3. The administration of mice with strain GDN1 through oral gavage for 25 days did not apparently change the host serum estradiol level of male mice. Serum estradiol level of mice was determined using the Estradiol Parameter Assay Kit (R&D Systems, Minneapolis, MN) after four weeks of the first oral administration. Results are representative of 3 individual experiments. Statistical results were calculated with unpaired *t*-test. Data shown are means ± SEM of 5~9 mice individuals (n = 5~9). Abbreviation: VEH, vehicle

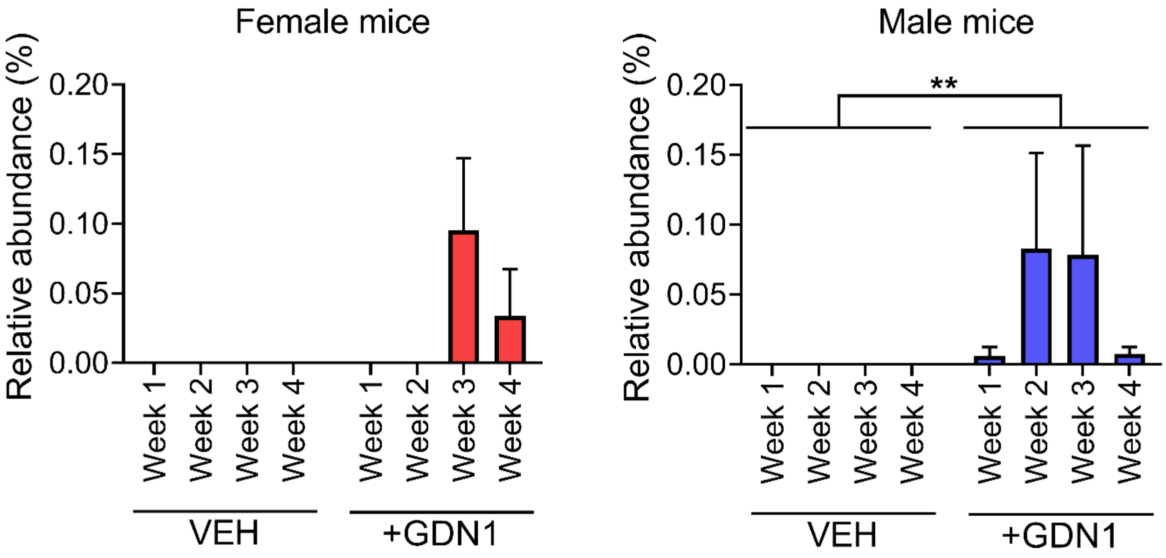

Fig. S4. Bacterial community structure analysis indicated the temporal changes in the relative abundance of *Thauera* in fecal samples. Bacterial 16S rRNA amplicons were sequenced on an Illumina platform. Week 1: fecal samples D0~D4; Week 2: fecal samples D7~D11; Week 3: fecal samples D14~D18; Week 4: fecal samples D21~D25. Statistical results were calculated with Wilcoxon rank sum test; $^{**}p < 0.01$. All data are shown as means ± SEM. Abbreviations: VEH, vehicle-administered mice; +GDN1, strain GDN1-administered mice.

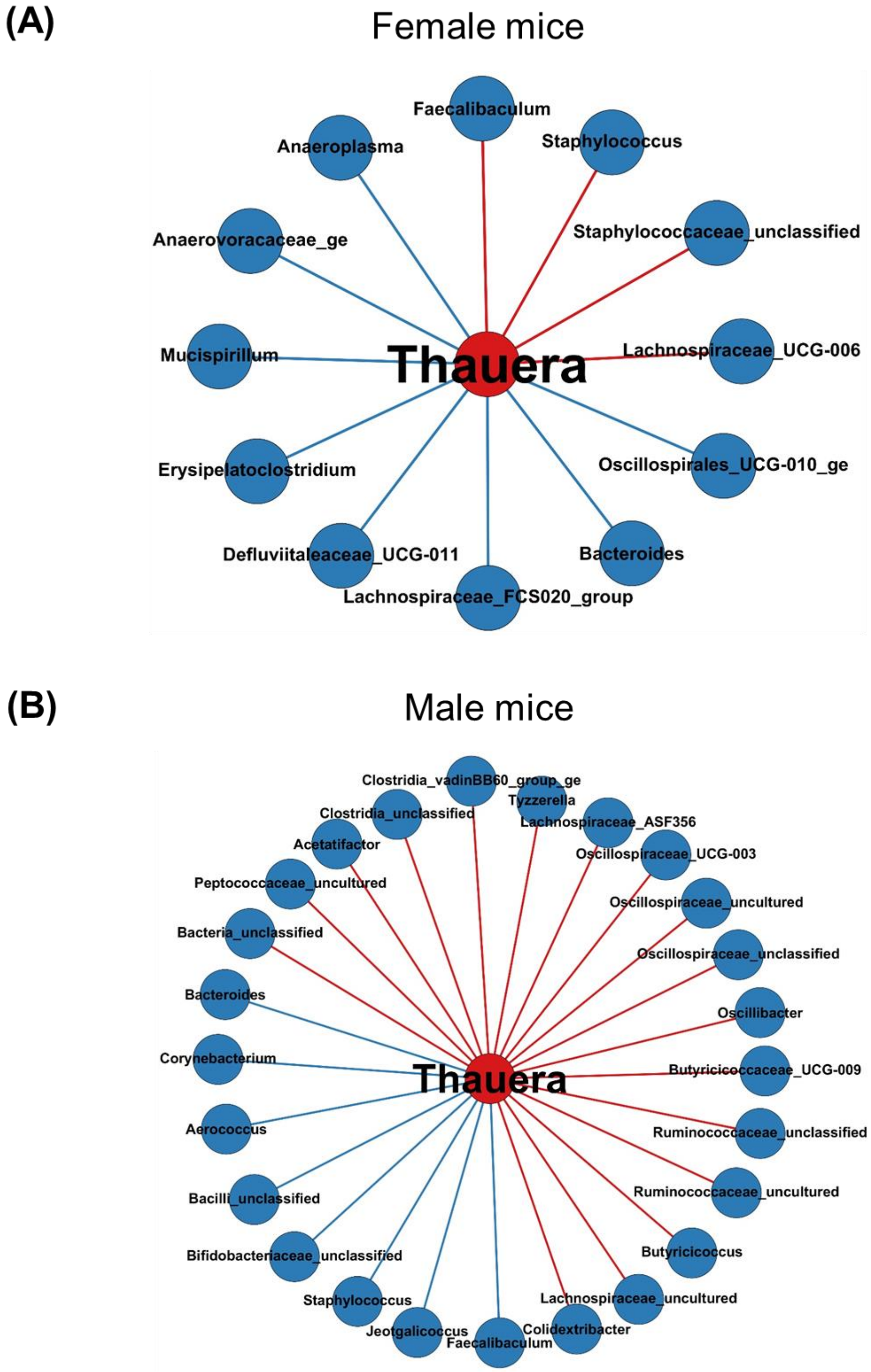

Fig. S5. The predicted interactions between *Thauera* and other gut microbes in the female (**A**) and male (**B**) mice. Microbial interactions were predicted using SparCC. Nodes represent microbial genera, and only high-scoring eigencentrality nodes are shown. A red edge depicts a positive correlation, while a blue edge depicts a negative correlation.

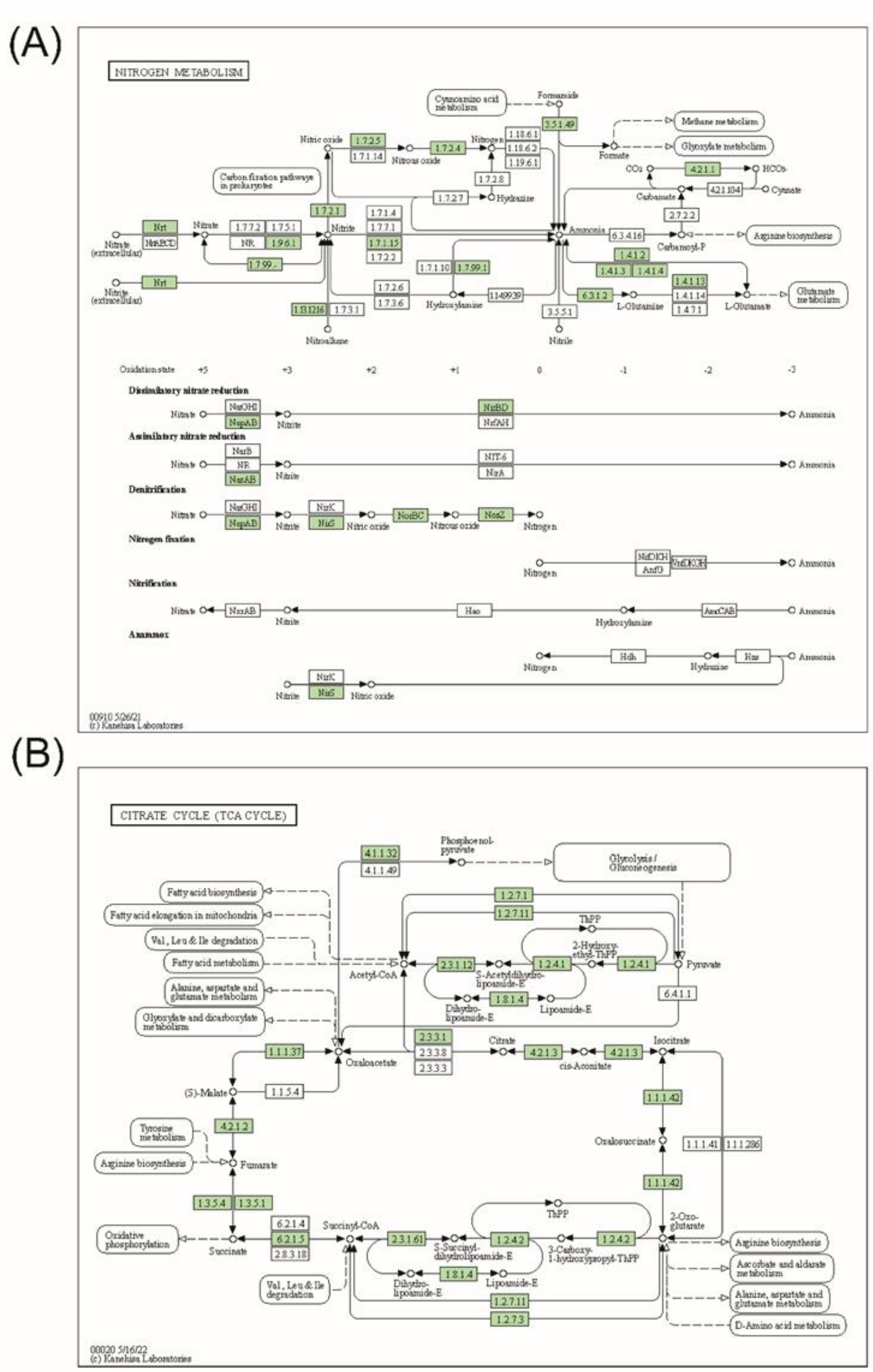

Fig. S6. KEGG (Kyoto Encyclopedia of Genes and Genomes) analysis revealed a complete set of genes participating in denitrification (**A**) and TCA cycle (**B**). Translated coding sequences of strain GDN1 was uploaded to KEGG and analyzed using the KofamKOALA tool with default parameters.

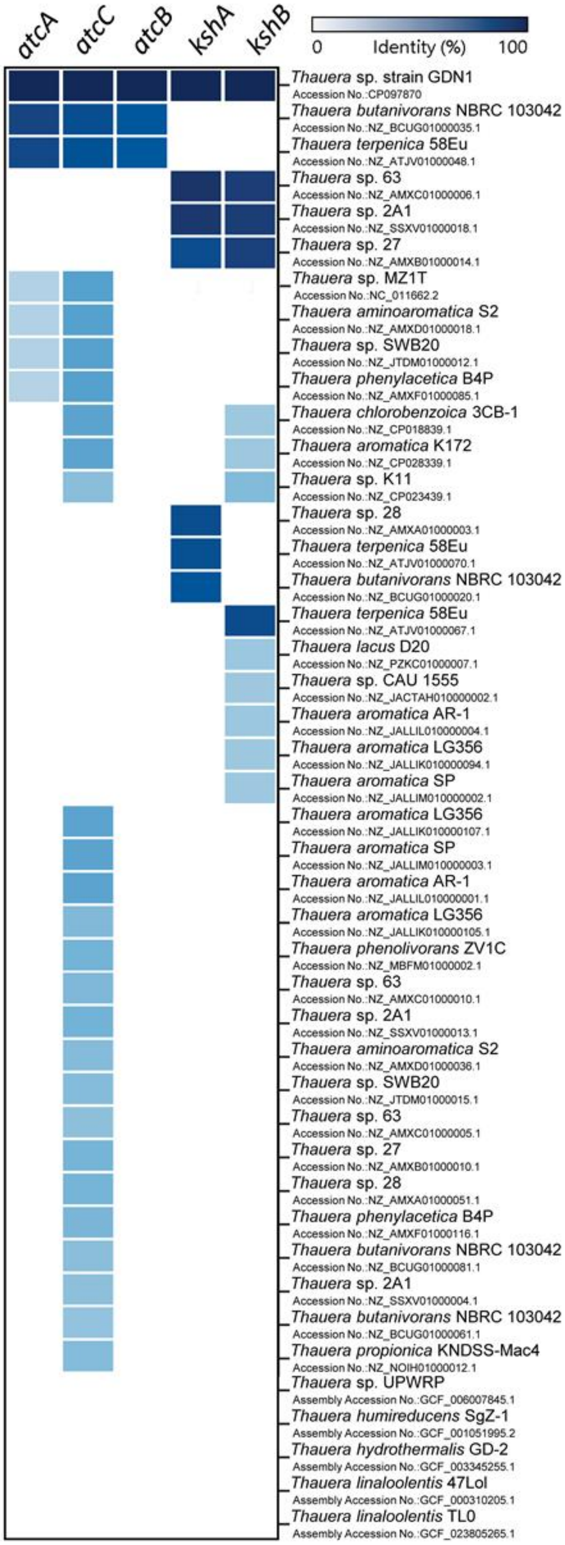

Fig. S7. Comparative genomic analysis of the *Thauera* isolates indicated the prevalence of androgen catabolic genes *atcABC* and *kshAB* in this genus. The homologous hit was set to 1 and sequence identity was larger than 30%.

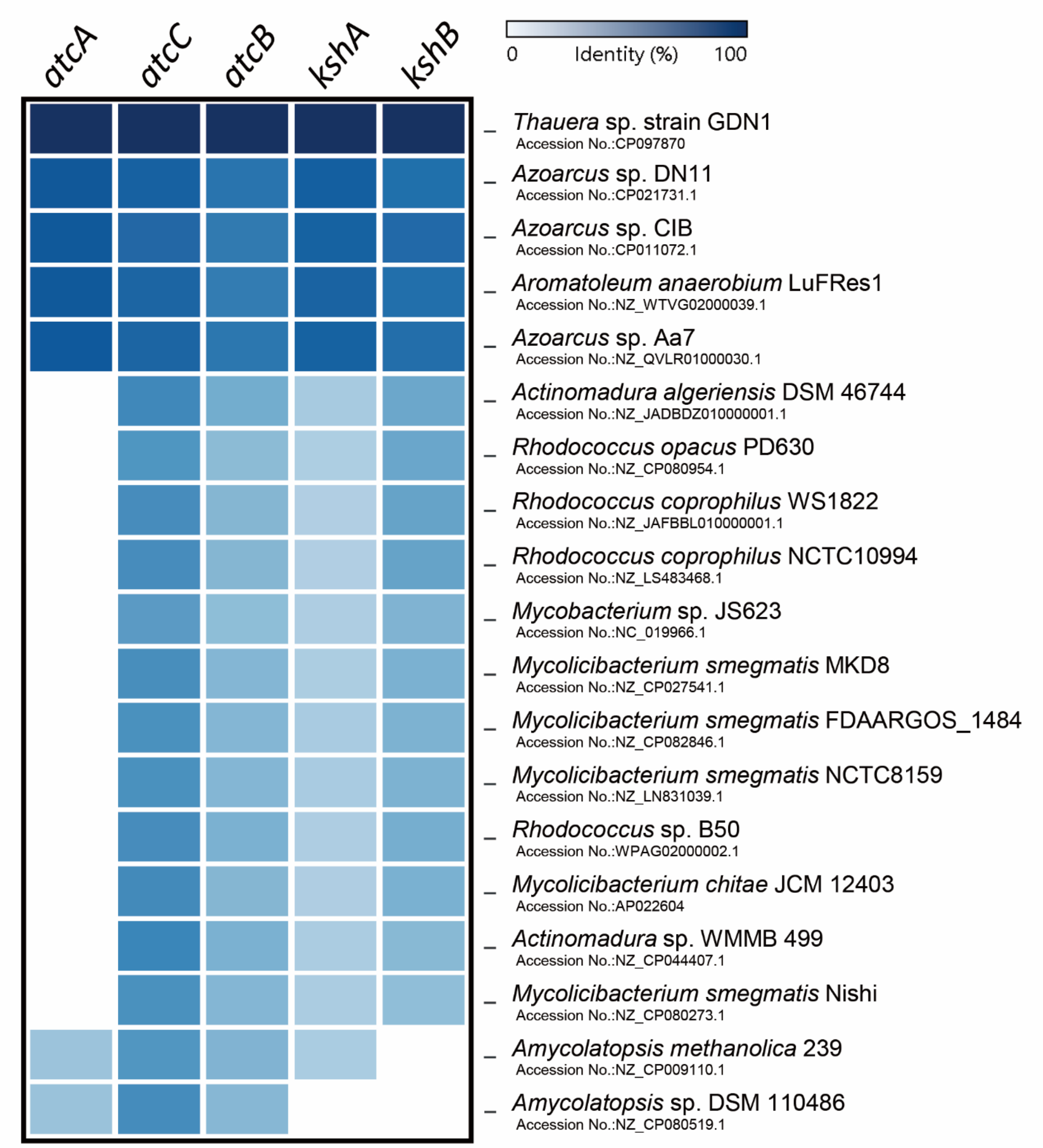

Fig. S8. Comparative genomic analysis of bacterial isolates indicated the prevalence of androgen catabolic genes *atcABC* and *kshAB* in other denitrifying proteobacteria. Note that the three androgen catabolic genera *Aromatoleum*, *Azoarcus*, and *Thauera* belong to the bacterial order Rhodocyclales. The primary protein structures derived from strain GDN1's androgen catabolic genes *atcABC* and *kshAB* were used as queries to search against all bacterial genomes available in the NCBI Nucleotide database. The homologous hit was set to 4 and sequence identity was larger than 30%.

Supplemental Tables

Table S1. Quality control of the RNA sequencing and the mapping status of clean reads.

Sample name	Anaerobic_T	Aerobic_T	Anaerobic_A	Aerobic_A
No. of raw read pairs	5,874,213	7,478,901	5,890,261	8,178,625
No. of clean read pairs	5,835,859	7,344,939	5,823,453	8,132,529
Total clean reads	11,671,918	14,689,878	11,546,906	16,265,058
Total mapped reads	11,575,353	14,121,307	11,548,277	16,095,777
Total mapping rate	99.17%	96.13%	99.15%	98.60%

The strain GDN1 transcriptomes were obtained from cells grown with acetate (abbreviation: A) or testosterone (abbreviation: T) under aerobic and anaerobic conditions.

Table S2. Oligonucleotide primers used in this study.

Primer	Sequence (5′- 3′)	Usage	Reference
q-GDN1-16S-F<br>q-GDN1-16S-R	ACCGGACTAAGAAGCACCG<br>AGCCTTGCAGTCACAAACG	Quantifying gene expression of strain GDN1-specific 16S rRNA	This study
q-GDN1-*atcA*-F<br>q-GDN1-*atcA*-R	GTGAGCACCTTCGTCGACT<br>CGCCGGTGTTTCGAGCA	Quantifying gene expression of strain GDN1-specific *atcA*	This study
q-GDN1-*kshA*-F<br>q-GDN1-*kshA*-R	GATCCTGCACACCATCACC<br>TGATCGAACCATCCTCGAC	Quantifying gene expression of strain GDN1-specific *kshA*	This study
q-universal-16S-F<br>q-universal-16S-R	AAACTCAAAKGAATTGACGG<br>CTCACRRCACGAGCTGAC	Quantifying gene expression of bacterial universal 16S rRNA	[74]
atcA-degenerate-F<br>*atcA*-degenerate-R	GGCASCGYYSAGTTCATCGACAA<br>GCCGCTGTCRTAYTCRTTSCCGCTSGG	Amplifying *atcA*-like genes from gut bacterial DNA	[30]
16S-341-F<br>16S-805-R	CCTACGGGNGGCWGCAG<br>GACTACHVGGGTATCTAATCC	Amplifying 16S rRNA (V3- V4 region) from fecal DNA	[75]

Table S3. Selection of housekeeping genes used as reference genes (black spots) in the global gene expression profiles of strain GDN1 (**Figure 4*B***).

**Locus tag** (CKCBHOJB_)	**Gene name**	**Definition**	**Aerobic_T [log$_2$(FPKM+1)]**	**Aerobic_A [log$_2$(FPKM+1)]**	**Anaerobic_T [log$_2$(FPKM+1)]**	**Anaerobic_A [log$_2$(FPKM+1)]**
00003	*gyrb*	DNA gyrase subunit B	6.90	7.28	7.59	6.72
00778	*ftsz*	Cell division protein FtsZ	9.33	9.48	9.07	9.08
00782	*sec1*	Protein translocase subunit SecA	6.21	8.23	7.84	8.05
00911	*rpob*	DNA-directed RNA polymerase (subunit β)	8.13	9.90	10.09	9.53
00912	*rpoc*	DNA-directed RNA polymerase (subunit β')	8.89	10.09	9.96	9.29
00944	*rpoa*	DNA-directed RNA polymerase (subunit α)	10.00	11.58	11.54	10.52
01269	*rho*	Transcription termination factor Rho	7.43	9.05	10.22	8.64
01304	*gyra*	DNA gyrase subunit A	7.26	8.53	8.31	8.15
01646	*sec2*	Protein translocase subunit SecA	8.66	8.22	7.51	8.30
02949	*dnag*	DNA primase	4.60	6.15	6.88	5.65
03234	*reca*	Protein RecA	7.73	8.86	8.87	9.26
03538	*gmk*	Guanylate kinase	5.19	7.03	6.20	6.62
00003	*gyrb*	DNA gyrase subunit B	6.90	7.28	7.59	6.72

The strain GDN1 transcriptomes were obtained from cells grown with acetate (abbreviation: A) or testosterone (abbreviation: T) under aerobic and anaerobic conditions.

Legends for Datasets

Dataset S1 (separate file). Genome annotation of strain GDN1 and transcriptomic analysis (RNA-Seq) of bacterial cells grown with testosterone or acetate under both aerobic and anaerobic conditions.

Dataset S2 (separate file). Eigenvector centrality values of individual bacterial genera in the gut microbiota of vehicle-administered and strain GDN1-administered female and male mice.

Appendices

Appendix S1. Nucleotide sequence of the 16S rRNA gene (CKCBHOJB _02702) of strain GDN1.

TTAAGAGTTTGATCCTGGCTCAGATTGAACGCTGGCGGCATGCTTTACACATGCAAG
TCGAACGGCAGCGGGGGCTTCGGCCTGCCGGCGAGTGGCGAACGGGTGAGTAATGC
ATCGGAACGTGCCCATGTCGTGGGGGATAACGTATCGAAAGGTACGCTAATACCGC
ATACGCCCTGAGGGGGAAAGCGGGGGATTCTTTGGAACCTCGCGCGATTGGAGCGG
CCGATGTCGGATTAGCTAGTAGGTGAGGTAAAGGCTCACCTAGGCGACGATCCGTA
GCGGGTCTGAGAGGATGATCCGCCACACTGGGACTGAGACACGGCCCAGACTCCTA
CGGGAGGCAGCAGTGGGGAATTTTGGACAATGGGCGCAAGCCTGATCCAGCCATGC
CGCGTGAGTGAAGAAGGCCTTCGGGTTGTAAAGCTCTTTCGGCCGGGAAGAAATCG
CGCGCTCTAACATAGCGCGTGGATGACGGTACCGGACTAAGAAGCACCGGCTAACT
ACGTGCCAGCAGCCGCGGTAATACGTAGGGTGCGAGCGTTAATCGGAATTACTGGG
CGTAAAGCGTGCGCAGGCGGTTTTGTAAGACAGATGTGAAATCCCCGGGCTTAACCT
GGGAACTGCGTTTGTGACTGCAAGGCTAGAGTACGGCAGAGGGGGGTGGAATTCCT
GGTGTAGCAGTGAAATGCGTAGAGATCAGGAGGAACACCGATGGCGAAGGCAGCC
CCCTGGGCCTGTACTGACGCTCATGCACGAAAGCGTGGGGAGCAAACAGGATTAGA
TACCCTGGTAGTCCACGCCCTAAACGATGTCGACTAGTCGTTCGGAGCAGCAATGCA
CTGAGTGACGCAGCTAACGCGTGAAGTCGACCGCCTGGGGAGTACGGCCGCAAGGT
TAAAACTCAAAGGAATTGACGGGGACCCGCACAAGCGGTGGATGATGTGGATTAAT
TCGATGCAACGCGAAAAACCTTACCTACCCTTGACATGTCTGGAACCTTGCTGAGAG
GCGAGGGTGCCTTCGGGAGCCAGAACACAGGTGCTGCATGGCTGTCGTCAGCTCGT
GTCGTGAGATGTTGGGTTAAGTCCCGCAACGAGCGCAACCCTTGTCACTAGTTGCCA
TCATTTAGTTGGGCACTCTAGTGAGACTGCCGGTGACAAACCGGAGGAAGGTGGGG
ATGACGTCAAGTCCTCATGGCCCTTATGGGTAGGGCTTCACACGTCATACAATGGTC
GGTACAGAGGGTTGCCAAGCCGCGAGGTGGAGCCAATCCCTTAAAGCCGATCGTAG
TCCGGATCGTAGTCTGCAACTCGACTACGTGAAGTCGGAATCGCTAGTAATCGCAGA
TCAGCATGCTGCGGTGAATACGTTCCCGGGTCTTGTACACACCGCCCGTCACACCAT
GGGAGTGGGTTTCACCAGAAGTAGGTAGCTTAACCTTCGGGAGGGCGCTTACCACG
GTGAGATTCATGACTGGGGTGAAGTCGTAACAAGGTAGCCGTATCGGAAGGTGCGG
CTGGATCACCTCCTTT

Appendix S2. Nucleotide sequence of the *atcA* gene (CKCBHOJB _02411) of strain GDN1.

ATGGCCATCGAAATTCCCACCCTGCCGGAACAGATGCCGAACGACGCCCCCGCTA
CCTCGGGAAGGCGGTCAACCGGGTCGAAGACCCCGCCCTCGTGTCGGGCACCGTCC
AGTTCATCGACAACCTCTCCCTGCCCGGCATGCTGCACTGCGCGATCCTGCGCAGTC
CGCATCCCCATGCCCGCATCCTGTCGATCAATGTGGACGCCGCGCGTGCCGCCGAAG
GCGTCGCCGCGGTACTCACTGGCGAGGACGTGCGGCGCTGGACCAACCCCTGCTTC
ACCGCCCCGGAAGGCTGGGGCAATTACTGCATGGCGGTAGACAAGGTGCGTTTCGT
CGGCGAGCCGGTCGTGGCCATCGCGGCGAGCAGCCGCTACCTGGCCGAGGACGCGC
TCGAACTGGTGGAAATCGAATACGAGCCGCTGGCGCCCGTCGCCAACCCCGAGCAG
GCGATGGCGCCCGGCGCGCCGGTCATTTTCGAGGAGCGCGGCACCAACGTCATGTT
GAGCCGCACCTACACCTGGGGCGAACTGGACCGTGTGTTCGCGGAAGCCGACCGCG
TGGTGAGCCGGCGCTTCCGCTGGAACCGCGTGGGCGCGAACCCCACCGAGACCTTC
GGCTGCATCTGCCAGTGGGACCTCGCCGACAACAGCCTCACCTGCCACGGCTCCTAC
CAGACACCCCGCTTCATGGCCATCGGCCGGGCCGCATCGCTGAACCTGCCGGCAAA
CCGCATCCGCATCGTCACCCACCCGCAGGGTGGCGGCTTCGGCGGCAAGGGCGGGC
CGCGCGGCACCGACATTGCCGCCCTGCTTTCGCGCAAGGCGCAGGGACGACCGGTC
AAGTACATCGAGGACCGCATGGAATACCTGCTCGCCGGCGGCGGGCAGTCCTGGGA
CCGCTATTACGACGCCGCGCTCGCGGTCAAGGCGGACGGCAGCGTCACCGGCTTCC
GTGTTCGTCTGGTGGACGACCAGGGCGCGGGCGCCGAGGGCTATGGAACGATCTCC
GCCGCGAAACCCCTGGCTGCGTTCACCGGCAACTATCGCATCGAGGCGGCCGGCTA
CGATCTGACCCTGGTCGCGACGAACCGTGCGCCGACCTACCCTTACCGCGGCTACGG
CCCTCCGCCGCACAACCTGGTGCTCGAGTCGCTGATGGACTGCACCGCGCGCGAACT
GGGCATCGACCCGGCCGAGCTGCGCCGGCGCAACTACATCCGCCCGGAACAGTTTC
CCTACACCGTGCCCAGCGGCAACGAATACGACAGCGGCAACTACGAGGCCGTGCTG
GATCGCGTCCTCGAGCTGGCCGACTACAAGGCCCTGCGTGCGCGCCAGGCCGAAGC
CCGCGCCCAGGGCAGGCTGGTCGGCATCGGCGTGGTCAATACCGTCGAGCCCGGCG
TCTTCGACTGGAACGCCTATGCCACCGTCGGCGTCCCCGGCGTCGGCGTGCCGGAAG
GCGTGAAGGTCGCGGTCGACCTGTTCGGCAACGTGACCGTGGCCGTGGGCTTCAAC
CTCCAGGGACAGGGGCAGTTCACCGTCGCCGCGCAGGTCGCAGCCGATTATTTCGG
CATCGACATGGCGCAGGTCCGCATCGCGAATACGCCCTCGGACGTGGCCCTGCCCC
ACTTCGGCCAGGGCGGCAGTCGCCTGGGCGTTGCCGTGACCGGCGCCACCCTTGGC
GCATGCGAAAAGCTCCGGGCGACCCTGTGCAAGGTCGCGGCGCACGTGATGCAGGC
GCCCGAGGATGCTGTGGCGCTGCGCAACGGCCGCCTGCACCGCATCGACGCACCCG
GGCATTCCATGTCGCTGGCCGAGATCGCGGGCCTGATGCTGTCGCGCACCGACCTAC
TGCCGGCAGGCGTCGAGCCCTGCCCGGAAGCGACCTACGTATGGGCCTCGCCCAAC
CGCAACGCGCCGGACGACCAGGGCCGCTGCCGCAGCTACCTGACCGCTGCCAACGC
CACCCATATCGCACTGATCGAAATCGATCGCGAAACCGGCCGCACCCACATCCTCG
ACTACGCCATCGTCGACGACTGCGGCACGCGCCTCAACCCGGCCAACGTGGAGGGC
CAGCTGCAGGGCGGCGTCGCCCAGGGCGTCGGCGCGGCATTGTACGAAGAGTACGT
CTACGACGCCGACTGCCAGCCGCTGGTGAGCACCTTCGTCGACTATCTGATCCCGAC
CATCCACGAAGTGCCGATGACGCGGAAGGATCACGTCGTCACGCCGTCGCCGGTCG
CACCGCTGGGCGCGAAGGGCTGTGGCGAAGGCGCGATCCACACCACGCCGGCGACC
ATCCTGTGTGCCATCAACGACGCCCTCGCGCCGCTTGGCAAGGAGCTGCTCGAAACA
CCGGCGTCGCCCCACCGCCTGTGGAAGCTGCTGCGCGAACATGACACTGCGGCTTG
A

Appendix S3. Nucleotide sequence of the *kshA* gene (CKCBHOJB _02279) of strain GDN1.

ATGGCCACAACGAAAGACTATCGCCTAGGCGAATACACCTTCCCGCGCGGCTGGTT
CATGATCGCCGAAGCGTCGGAACTGGATACCCACAAGCCGCACGCGGTGCGCTTCT
TCGGCCAGGACTTCGCGCTCTACCGCGGCCGCGAGAGCGGCAAGGTGGTGCTGCTC
GACGCCTACTGCCCGCACATGAAGACCCACCTCGCCGCGCCCAACAAGACCTCGTA
CGTGGTGCTCGACGGCGGCGGCACCAACGTCGAAGGTGACGGCATCCGCTGCCCCT
ACCACGGCTGGCGCTTCGGTGCCGACGGCAAGTGCGACCACATCCCCTACCACGAA
GGCCAGATCCCGGCCGCAGCCAAGGTGAAGTCGTGGCCGGTGGTCGAGAGCCTGGG
GGCGATCTGGGTGTGGCACGACCCGGAAGGCGGCGAACCGGAATGGGATCACCCCA
TGCTCGCTGAATGGAACGACCCGGCCTGGGTGCACTGGAAGTTCGACCACCTCGGC
ATGCTCAACCAGCACCCGCAGGAAGTCATCGACAACATCTGCGACTACGGCCACCT
GAGCCCGATCCACGGCTCGACCGTGCTCAAGTACGAGAACGAGTTCCAGGGCCACA
AAGCGATCCAGCGCCAGTGCGGCCCGCACCGCACCCTGGTCGGTGAGGACGGCGTC
AGCCCGATCCTGCACACCATCACCATGTACCACGGCCCTGGCGTGCTGATCTCGCAC
CTCACCGGCCTGTACGACGCCGTGATGATGATCTGCAACACCCCGGTCGAGGATGGT
TCGATCAAGGTCTGGCACGCCCTGCTGGTGAAGTCGCCGAGTGGCAGCAAGGTGGC
GACCCACACCGACATGGTGGCAGCGCGCCACTATCAGGAGATGGCACTCACCGCCT
TCGCCCAGGACTTCGAGGTGTGGTCGCACAAGGCGGCCTGCCTGAACGGGCTCTTCA
TCCCGAGCGACGGCCCGTTCATGAAGGCGCGCATCTGGTACAAGCAGTTCTACAAC
CCGCGTGCGAAGAAGAACGAATACCTCGAGCAGTGCGAGGGCTACTACGTGCCGAA
GGGGATTGCGCCTTACACCGACGAGCCTGTCGCCGCCTGA

Appendix S4. Nucleotide sequence of the inherent *atcA*-like DNA fragment extracted from the vehicle mouse caecum.

GGCAGCGTCCAGTTCATCGACAAGTATGCCTTCCAGTGTGGATATTGCACCCCAGGC
ATCATCATGTCGGCCCGGGCGCTGCTGGATAAGAACCCGCATCCCACGAAGGAGGA
GATTGGTGAAGCATTAGCGGGCAACTTCTGCCGCTGTATCAGCCACTACCATGTATT
TGAAGCAGTGGAGCAGGTTGCTGCTGAAGGGAGGTAGCTCATGGAAAAAACGGATA
TGATCGATAAGAAGGAATATCGTCATATAGGAAAGTATGTTCCCAGGAAGGATGCC
CGTGACATCGTCACCGGCAAGTGCGTGTATCTGGAGGACCACAAGTTTGATAACCTT
CTCTATGCGCGGGTGATCGGCAGCCCCTATGCCCACGCCATGATCAAGAAGATCGA
CACTAGCAAGGCGGAGGCCCTGCCCGGCGTGGCCGCTGTAGTCACCTATAAGAACC
AGCCCGAGTGGTCCAAACGGTTCTTACAGGGTACGCCGCCCACAAAGCCCTTGCTAT
CCCAGCAGGTCCGGATGGTGGGCGACCCGATCGCCCTGGTGGCCGCTGAGACGGAG
GATCTCTGCCGGCAGGCGGGACGCCTGATCGAGGTGGAGTATGAGCAGCTGGCGGC
GGTGTTCGATCCTATGAAGGCCATGGAGCTGGACGCTCCCAGGATCTATCATGAATT
TGAGACCAACCGCTGGCCTAAGGACGACGTGGCCTTCGGCGACGAGCGGATGCTGG
CTTCCGTGGAGCGGGGAGATGTGGATAAGGAATTTGCCGAGGCCGACTTTATCGGC
GGCGGCGTCTGCGGCTTTGAGACCAGGACGGTGCCCTTAGCCCCCGAGCCGCCCAG
CGGCAATGAGTACGACAGCGGCAGATCTGGATCCCCTCTAGAGTCGA
